# Grading the evidence for sex‐specific VO_2_max adaptation: An evidence‐stratified narrative review of central, peripheral, and pulmonary determinants

**DOI:** 10.14814/phy2.71157

**Published:** 2026-11-09

**Authors:** Alireza Rashidpour, Elaheh Piralaiy, Saeid Nikoukheslat

**Affiliations:** ^1^ Department of Exercise Physiology, Faculty of Physical Education and Sport Sciences University of Tabriz Tabriz Iran

**Keywords:** arteriovenous oxygen difference, cardiac output, cardiorespiratory fitness, maximal oxygen uptake, pulmonary diffusion, sex differences

## Abstract

Maximal oxygen uptake (VO_2_max) strongly predicts all‐cause mortality, yet the step limiting it shifts along the oxygen transport cascade, and women remain underrepresented in the literature underpinning exercise guidelines. This narrative review integrates the mechanistic determinants of VO_2_max, examines how the rate‐limiting step shifts with training status, modality, environment, and biological sex, and—as its principal contribution—systematically grades the evidence for sex‐specific adaptation as established, emerging, or speculative. Convective oxygen delivery predominates in untrained individuals, whereas a substantial proportion of elite athletes develop exercise‐induced arterial hypoxemia (EIAH) reflecting pulmonary diffusion limitation. Women's lower absolute VO_2_max is largely explained by blood volume and oxygen‐carrying capacity. Sex differences in the magnitude of central cardiac remodeling are meta‐analytically supported, but the proposed time‐course difference rests on a single small cohort (*n* = 12), and peripheral‐dominant adaptation derives from limited samples. Recent direct comparisons of EIAH between sexes conflict, and menstrual‐cycle phase has at most a small effect on aerobic performance. Where evidence is strongest, sex‐stratified interpretation is defensible; where weakest, individualized approaches remain a research direction. Adequately powered, sex‐stratified, methodologically standardized studies are the field's most pressing need.

## Graphical abstract

The step limiting VO_2_max is context‐ and sex‐dependent. Evidence supports sex‐stratified standards and individualized, adaptation‐monitored training—not fixed sex‐based rules.

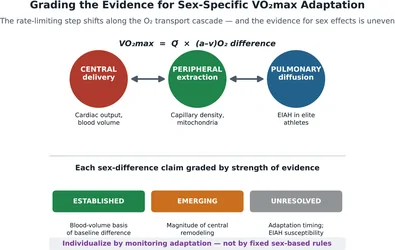

## 1 INTRODUCTION

Maximal oxygen uptake quantifies the integrated capacity of the respiratory, cardiovascular, and muscular systems to take up, transport, and use oxygen, and remains among the strongest physiological predictors of all‐cause mortality (Kokkinos et al., 2022; Mandsager et al., 2018). The question of what limits VO_2_max has been debated since Hill and Lupton (1923) proposed a central cardiovascular ceiling, and persists partly because it has often been framed as a search for a single answer (Bassett & Howley, 2000; Wagner, 1996). A more tractable framing, developed here, is that the rate‐limiting step is not fixed but moves along the oxygen transport cascade with training status, exercise modality, environment, age, and biological sex.

Although women constitute roughly half of recreational endurance participants (Besson et al., 2022), they are underrepresented in the evidence base: in a survey of 5261 publications across six leading journals women constituted approximately 34% of participants and only 6% of studies enrolled women exclusively (Cowley et al., 2021), a gap associated with the gender of authorship and editorial composition (Costello et al., 2014; James et al., 2023). This representation gap and its potential downstream consequences are summarized in Figure 1. Training recommendations derived predominantly from young men are nonetheless applied universally, and extrapolation of male‐centric physiology to women is not universally appropriate (Hunter & Senefeld, 2024). Sex differences in endurance performance and its determinants are now well documented across running and Olympic winter endurance disciplines (Besson et al., 2022; Solli et al., 2024). There are also biologically plausible reasons—hormonal milieu, cardiac and vascular structure, substrate metabolism—to expect that adaptation to a given stimulus may differ between sexes (Diaz‐Canestro & Montero, 2019; Howden et al., 2015; Hunter et al., 2023). Whether these differences are large enough, and sufficiently characterized, to warrant sex‐specific prescription is the central empirical question we examine.

**FIGURE 1:**
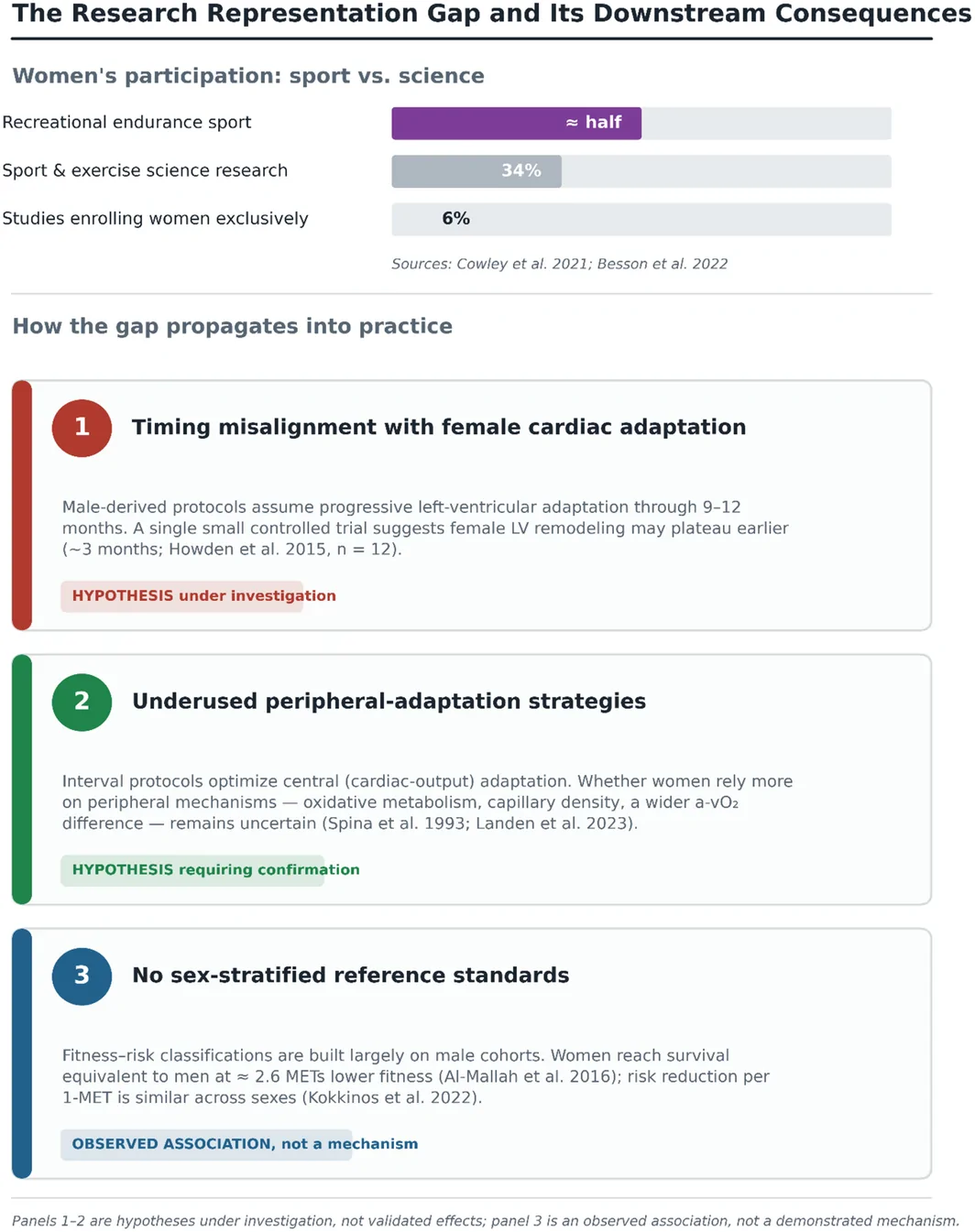
The research representation gap and its downstream consequences. Although women constitute approximately half of recreational endurance participants, they account for only ~34% of participants in sport and exercise science research, with just 6% of studies enrolling women exclusively (Cowley et al., 2021). The figure depicts how this representation gap propagates into three candidate consequences requiring empirical confirmation rather than established facts: (1) potential misalignment of male‐derived adaptation timelines with female cardiac remodeling, (2) possible underutilization of peripheral‐adaptation strategies, and (3) absence of sex‐stratified reference standards despite women achieving equivalent survival at approximately 2.6 METs lower absolute fitness (Al‐Mallah et al., 2016). Panels 1–2 represent hypotheses under investigation, not validated effects; the fitness‐survival contrast (panel 3) depicts an observed association, not a demonstrated mechanism.

This review has four aims. First, we synthesize the mechanistic determinants of VO_2_max through the Fick framework and characterize how the rate‐limiting step shifts with training status, modality, environment, and sex. Second—and as the review's principal contribution—we systematically grade the evidence for each major sex‐difference claim as established, emerging, or speculative, thereby separating conclusions that can currently guide practice from those that remain hypotheses. Third, we consider the practical implications of these graded findings for the interpretation of VO_2_max reference standards. Fourth, we examine the methodological constraints that generate this unevenness and identify the research priorities required to resolve it. This evidence‐stratified approach distinguishes the present review from prior overviews of sex differences in VO_2_max (Santisteban et al., 2022), which have synthesized findings without systematically appraising the strength of the evidence behind each claim.

### 1.1 Scope and approach

This is a narrative, not a systematic, review. The literature was identified through structured PubMed searches combining terms for the oxygen transport cascade (VO_2_max, cardiac output, stroke volume, hemoglobin mass, arteriovenous oxygen difference, pulmonary diffusion, exercise‐induced arterial hypoxemia) with terms for biological sex and adaptation (sex differences, female, menstrual cycle, cardiac remodeling, trainability), prioritizing work published between 2015 and 2026 while retaining foundational earlier sources that established the mechanistic framework. Sources were selected for relevance to the mechanistic and sex‐comparative focus of the review rather than through formal systematic screening or quantitative pooling; accordingly, the synthesis is interpretive and evidence‐graded rather than meta‐analytic. To make the strength of evidence explicit, key claims are labeled throughout as established (consistent support from multiple studies or meta‐analyses), emerging (supported but requiring replication), or speculative/unresolved (limited or conflicting evidence), and are summarized on this basis in Table 1.

**TABLE 1:** Evidence grading for key claims regarding sex‐specific VO_2_max adaptation.

Claim	Evidence level	Principal support	Key limitation
Lower absolute VO_2_max in women, largely explained by blood volume/O_2_‐carrying capacity	Established	(Diaz‐Canestro et al., 2022b; Diaz‐Canestro & Montero, 2022; Kontro et al., 2024; Webb et al., 2023)	Residual variance after scaling not fully resolved
Cardiac‐to‐pulmonary shift in limitation with rising fitness	Established	(Dempsey & Wagner, 1999; Mandić et al., 2023; Powers et al., 1989, 1993)	EIAH cohorts skew male
Greater magnitude of central (LV, Q̇, SV) adaptation in men	Emerging	(Astorino et al., 2022; Cotie et al., 2025; Diaz‐Canestro & Montero, 2019)	Protocol/baseline heterogeneity
Earlier female LV plateau (~3 vs. 9–12 months)	Emerging (single small cohort)	(Howden et al., 2015) (*n* = 12; 7 M, 5 F; CMR + PA catheterization)	Single trial, one protocol, small and sex‐unbalanced sample
Peripheral‐dominant adaptation in women	Speculative → emerging	(Ferri‐Marini et al., 2026; Landen et al., 2023; Spina et al., 1993)	Small/cross‐sectional samples
Greater female EIAH susceptibility	Unresolved (conflicting)	(Harms et al., 1998; Hind et al., 2025)	No adequately powered, size‐controlled comparison
Menstrual‐cycle phase materially affects VO_2_max	Low/small effect	(Colenso‐Semple et al., 2023; D'Souza et al., 2023; McNulty et al., 2020; Schumpf et al., 2023)	Trivial pooled effect sizes

*Note*: Evidence levels are defined as follows: established = consistent support from multiple studies or meta‐analyses; emerging = supported but requiring replication; speculative/unresolved = limited or conflicting evidence.

Abbreviations: CMR, cardiac magnetic resonance; EIAH, exercise‐induced arterial hypoxemia; F, female; LV, left ventricular; M, male; PA, pulmonary artery; Q̇, cardiac output; SV, stroke volume; VO_2_max, maximal oxygen uptake.

## 2 PHYSIOLOGICAL DETERMINANTS OF VO_2_max

VO_2_max is described by the Fick principle, VO_2_ = Q̇ × (Ca–Cv̄)O_2_, where cardiac output (Q̇) is the convective component and the arteriovenous oxygen content difference [(Ca–Cv̄)O_2_] reflects both arterial oxygen content and fractional extraction. Each term decomposes into a chain of conductances—ventilation and pulmonary diffusion, hemoglobin mass and oxygen‐carrying capacity, stroke volume and heart rate, regional perfusion and capillary exchange surface, and mitochondrial oxidative capacity—operating largely in series (Wagner, 1993, 1996). Because the steps are in series, the ceiling is set by whichever conductance is least adequate for a given demand, which is why the limiting step is context‐dependent.

### 2.1 Convective delivery and oxygen‐carrying capacity

Cardiac output is the largest single contributor to interindividual VO_2_max variance; stroke volume is its trainable component, whereas maximal heart rate is largely fixed and declines with age. The convective term is itself constrained by hemoglobin mass: a systematic review and meta‐analysis confirms a strong positive relationship between total hemoglobin and VO_2_max across the fitness spectrum (Webb et al., 2023), and arterial oxygen saturation modulates this relationship (Goodrich et al., 2018). Experimentally raising oxygen‐carrying capacity increases VO_2_max, whereas pharmacologically blunting the rise in heart rate during exercise (impairing chronotropic competence, the ability of the heart to increase its rate appropriately with exertion) lowers it (Ekblom et al., 1972; Tesch & Kaiser, 1983); both effects confirm that oxygen delivery is rate‐limiting under whole‐body conditions in most individuals.

### 2.2 Sex, blood volume, and the basis of the baseline difference

The 20%–30% lower absolute VO_2_max in women is substantially explained by differences in blood volume and oxygen‐carrying capacity rather than by an intrinsic cardiac deficit. In integrative physiological studies, differences in cardiac output and aerobic capacity between sexes are largely accounted for by blood volume and hemoglobin (Diaz‐Canestro et al., 2022b; Koons et al., 2019), and these volumetric determinants themselves change with age (Diaz‐Canestro & Montero, 2022). Experimental removal of a defined volume of blood (acute blood withdrawal, which lowers blood volume) impairs cardiac filling, output, and aerobic capacity in proportion to the induced reduction in blood volume (Diaz‐Canestro et al., 2022a). When hematological values are normalized to fat‐free mass in individuals matched for aerobic fitness, some—but not all—sex differences attenuate (Kontro et al., 2024), indicating that body‐size scaling is necessary but not always sufficient to explain the disparity. These contemporary studies supersede reliance on the earlier, smaller demonstration by Wiebe et al. (1998). which compared exercise cardiac function between endurance‐trained men and women and was among the first to attribute the sex difference in aerobic capacity partly to blood volume.

### 2.3 Diffusive and extractive components

At the muscle, oxygen offloading depends on capillary density (transit time, surface area) and the mitochondrial sink. Isolated small‐muscle‐mass models achieve fractional extraction exceeding 90% (Andersen & Saltin, 1985), and mitochondrial respiratory capacity exceeds maximal whole‐body oxygen delivery (Boushel et al., 2011), demonstrating large peripheral reserve when delivery is not limiting. The periphery thus becomes relevant where delivery is adequate or central adaptation has plateaued.

### 2.4 Pulmonary component

In most people the lung is overbuilt for exercise and arterial saturation is maintained to exhaustion; this ceases to hold at the upper extreme of cardiac output (Section 3).

### 2.5 Hormonal and autonomic modulation

Testosterone supports erythropoiesis, cardiac protein synthesis, and skeletal‐muscle anabolism (Szabó et al., 2018), contributing to larger absolute cardiac and blood‐volume capacities in men (that is, higher values before scaling for body size). Estrogen promotes VEGF‐mediated angiogenesis and nitric‐oxide–dependent vasodilation (Hellsten & Nyberg, 2016; Moreau & Hildreth, 2014), and premenopausal women show greater arterial compliance and lower afterload than age‐matched men (Parker et al., 2010). Autonomic regulation sets maximal heart rate and the kinetics of cardiac filling and is itself modifiable by training, contributing to interindividual variability in the oxygen‐delivery (convective) response. These mechanisms are well established at the level of physiology; their quantitative translation into differential trainability is where evidence thins (Sections 3, 4).

## 3 CONTEXT‐DEPENDENT LIMITATIONS OF VO_2_max

### 3.1 Training status: The cardiac‐to‐pulmonary shift (established)

In untrained individuals, convective delivery predominates: raising hemoglobin concentration produces near‐proportional VO_2_max gains (Calbet et al., 2006; Ekblom et al., 1972), β‐blockade lowers VO_2_max via reduced maximal heart rate (Tesch & Kaiser, 1983), and peripheral reserve is large (Andersen & Saltin, 1985). That central delivery drives the trainable response even to brief, intense protocols is now demonstrated with gold‐standard methods: improvements in VO_2_max after sprint‐interval training coincide with, and are mediated by, increases in central hemodynamic factors (cardiac output and its determinants, stroke volume and heart rate) confirmed by right heart catheterization (Mandić et al., 2022, 2023), and meta‐analysis identifies central cardiovascular factors as principal contributors to interval‐training–induced VO_2_max gains (Astorino et al., 2022; Rosenblat et al., 2022).

At the opposite extreme, roughly 40%–50% of athletes with VO_2_max >70 mL·kg^−1^·min^−1^ develop exercise‐induced arterial hypoxemia (EIAH)—arterial desaturation below ~93%–95% at maximal effort (Dempsey & Wagner, 1999; Powers et al., 1993)—reflecting diffusion limitation at very high cardiac outputs, with contributions from shortened red‐cell pulmonary transit time (Warren et al., 1991), ventilation–perfusion heterogeneity (Hopkins et al., 1998), and alveolar–capillary diffusion limitation. The strongest causal evidence is interventional: preventing desaturation with mild hyperoxia restores VO_2_max by several percent in affected athletes (Powers et al., 1989). EIAH is recognized to occur in fit individuals of both sexes and across ages (Dempsey & Wagner, 1999).

### 3.2 Exercise modality (established)

Whole‐body exercise loads central delivery, whereas small‐muscle‐mass exercise shifts limitation peripherally to local perfusion and oxidative capacity (Saltin & Strange, 1992; Secher et al., 1977). Domain‐specific training studies confirm that the central/peripheral balance of adaptation depends on the exercise stimulus itself (Inglis et al., 2025). An athlete's limiting factor in sport‐specific work may therefore differ from that measured in a laboratory whole‐body test.

### 3.3 Environment (established to emerging)

Altitude raises hemoglobin via hypoxia‐driven erythropoiesis but elevates viscosity; beyond roughly 50% hematocrit, delivery gains from additional red‐cell mass are offset by reduced flow (Brun et al., 2000; Calbet et al., 2006)—an instructive case in which optimizing one conductance creates a bottleneck elsewhere. Heat stress diverts blood to the skin, reducing volume available to working muscle and imposing an effective delivery limitation (Périard et al., 2015).

### 3.4 Integrated model

The rate‐limiting step is best understood as a moving target: convective delivery in the untrained, pulmonary diffusion in the elite, arterial content at altitude, and a shifting central/peripheral balance with modality and age (Inglis et al., 2025; Lanza et al., 2008). Whether biological sex systematically affects where along this cascade the limitation sits is examined next. The evidence there is weaker than for the contextual factors above.

## 4 EVIDENCE FOR SEX‐SPECIFIC ADAPTATION PATTERNS

This section separates established from emerging and speculative claims; Table 1 summarizes the grading.

### 4.1 Baseline differences (established)

As developed in Section 2, women's lower absolute VO_2_max is largely attributable to blood volume and oxygen‐carrying capacity, with residual variance after body‐size scaling (Diaz‐Canestro et al., 2022b; Kontro et al., 2024; Santisteban et al., 2022).

### 4.2 Magnitude of central adaptation (emerging)

A systematic review and meta‐analysis reports greater training‐induced gains in left‐ventricular volume, peak cardiac output, and stroke volume in men than women (Cotie et al., 2025). A hemodynamic study found sex differences in the response to high‐intensity interval exercise, although peak relative VO_2_, Q̇, and stroke volume did not differ when expressed relative to body size (Coe & Astorino, 2024). Meta‐analysis indicates sex dimorphism in VO_2_max trainability favoring men (Diaz‐Canestro & Montero, 2019), and sex‐specific patterns of cardiorespiratory fitness change after training are observed even in clinical populations (Vidal‐Almela et al., 2024). Direct echocardiographic evidence shows measurable left‐ventricular remodeling after as little as 6 weeks of sprint‐interval training (Eriksson et al., 2024), and lifelong endurance exercise meaningfully shapes cardiovascular structure and function in women (Carrick‐Ranson et al., 2020). These converging analyses move the magnitude claim from speculative to emerging, though heterogeneity in protocols and baseline training status limits firm quantitative conclusions.

### 4.3 Time course of central adaptation (emerging; single small cohort)

The widely cited contrast—LV mass plateauing at ~3 months in women versus progressive gains through ~12 months in men—derives principally from Howden et al. (2015), a methodologically rigorous but very small controlled trial (*n* = 12; 7 men, 5 women) that employed cardiac magnetic resonance imaging and pulmonary artery catheterization to characterize the longitudinal cardiovascular response to 1 year of matched endurance training. The use of gold‐standard imaging and invasive hemodynamics lends the finding internal credibility; however, the small sample, single protocol, and unequal sex distribution mean it cannot yet support timeline‐based prescription and requires replication across protocols and populations. We therefore present it as a hypothesis‐generating observation rather than as an established kinetic law.

### 4.4 Peripheral and metabolic adaptation (speculative to emerging)

The proposal that women compensate via peripheral mechanisms rests partly on Spina et al. (1993) (*n* = 9 older women) and is therefore hypothesis‐generating only. It is mechanistically plausible: contemporary multi‐omic work demonstrates sex differences in skeletal‐muscle protein expression and DNA methylation in response to training (Landen et al., 2023), and meta‐analysis confirms sex differences in fat oxidation that depend on age, body mass, and modality (Ferri‐Marini et al., 2026; Roepstorff et al., 2002; Tarnopolsky, 2008). Cross‐sectional data suggest a meaningful extraction contribution to VO_2_max (Skattebo et al., 2020). However, “women are peripherally limited” is not an established generalization and should not be stated as one.

### 4.5 EIAH and sex (unresolved)

Earlier work documented EIAH in healthy young women, with smaller lung volumes and diffusion surface area relative to height‐matched men offering a plausible mechanism for greater susceptibility (Harms et al., 1998). However, a recent direct comparison in trained endurance athletes found that all participants developed EIAH with no sex difference in end‐exercise SpO_2_ (89% men vs. 90% women) and no pulmonary edema in either sex (Hind et al., 2025). The balance of evidence is, therefore, genuinely conflicting, and a sex difference in EIAH at matched relative intensity is not established.

### 4.6 Synthesis

Sex plausibly modulates the magnitude—and possibly the time course—of central remodeling and the relative reliance on peripheral mechanisms; the magnitude claim is best supported (meta‐analytic), the timeline claim rests on one small cohort, and the peripheral‐dominance and EIAH claims remain unresolved.

## 5 METHODOLOGICAL CHALLENGES IN INTERPRETING SEX DIFFERENCES

The unevenness above is partly a function of how the literature was generated, and any rigorous review must foreground these constraints.

### 5.1 Underrepresentation and analytic practice

Beyond low female enrollment (~34%) (Costello et al., 2014; Cowley et al., 2021), many studies that include women pool the sexes or are underpowered for sex‐stratified analysis, so claims of “no sex difference” are frequently uninterpretable. The underrepresentation of female participants is correlated with the proportion of women among study authors and journal editors—studies and journals with more women in these roles tend to enroll more female participants (James et al., 2023). This deficit extends to specific subfields: an audit of the vascular‐adaptation literature found persistent female underrepresentation (Thompson et al., 2024).

### 5.2 Menstrual cycle and hormonal status

Within‐cycle hormonal fluctuations modulate substrate use and thermoregulation, but recent systematic reviews and meta‐analyses indicate the effect on aerobic and strength performance is small to trivial: a network meta‐analysis of 73 studies reported trivial effect sizes across cycle phases (Colenso‐Semple et al., 2023; McNulty et al., 2020), and a dedicated meta‐analysis found limited and inconsistent effects of cycle phase and hormonal contraceptives on VO_2_max (Schumpf et al., 2023). A multimethod systems‐physiology review further documents that hormonal contraceptives introduce a distinct, often unreported, exogenous hormonal state (D'Souza et al., 2023), and menstrual blood loss and contraceptive use can affect oxygen‐carrying capacity itself (Keller et al., 2020). The principal implication is methodological—phase and contraceptive status should be documented and standardized—rather than a basis for sex‐divergent prescription (Elliott‐Sale et al., 2021).

### 5.3 Training‐status mismatching

Studies frequently compare women and men described as similarly active who have nonetheless accumulated different absolute training volumes and intensities. Because the magnitude of adaptation depends on the training stimulus received, this confounds “sex” with “dose”: an apparent sex difference in trainability may instead reflect the two groups occupying different positions on the dose–response curve. Rigorous inference therefore requires matching participants on relative training load, not merely on activity labels (Elliott‐Sale et al., 2021).

### 5.4 Responder/non‐responder classification

Because individual physiological adaptations show limited reproducibility across repeated identical training periods, a substantial fraction of apparent “non‐responders” reflects within‐person variability and measurement error rather than fixed biology (Bouchard et al., 2011; Odden et al., 2026); differential variance between sexes can therefore distort responder‐rate comparisons.

### 5.5 Sample size, measurement error, and publication bias

Several pivotal sex‐comparison findings derive from very small cohorts: the central‐adaptation time‐course contrast rests on 12 participants (Howden et al., 2015) and the peripheral‐dominance hypothesis on 9 older women (Spina et al., 1993). With VO_2_max measurement error of ~4%–6% and unequal sex distributions, such studies are well suited to hypothesis generation but cannot establish kinetic timelines or prescriptive thresholds. The contrast between the methodological rigor of these studies (e.g. invasive hemodynamics and cardiac imaging in Howden et al.) and their limited statistical power is precisely why their findings should anchor future adequately powered trials rather than current prescription.

These constraints do not negate sex effects; they bound the confidence with which any single quantitative claim—especially a numeric prescription threshold—can be advanced.

## 6 TRANSLATIONAL IMPLICATIONS

The robust translational point is interpretive, not prescriptive: VO_2_max reference standards built predominantly on male cohorts may misclassify women, because a given absolute VO_2_max corresponds to different relative fitness between sexes (Kaminsky et al., 2013; Ross & Myers, 2023)—women reaching equivalent all‐cause survival at approximately 2.6 METs lower fitness (Al‐Mallah et al., 2016). Sex‐stratified normative data are therefore a defensible, low‐risk recommendation. Mechanistic claims of female “cardioprotection” are not yet warranted from these associations.

By contrast, modality‐ and timing‐specific training recommendations by sex are not yet established. Two special cases warrant explicit mention. First, women in clinical rehabilitation may be under‐dosed: a randomized trial demonstrated that optimizing training intensity improves the cardiorespiratory response in women in cardiac rehabilitation (Khadanga et al., 2022). Second, the menopause transition modifies adaptation—menopause alters the aerobic response to high‐intensity interval training (Nio et al., 2020), exercise lowers blood pressure in postmenopausal women (Debray et al., 2023), and vascular health across the female lifespan (puberty, pregnancy, menopause) is a distinct trajectory requiring tailored study (Miller et al., 2025). Interval training is efficacious for cardiometabolic health in reproductive‐aged women (Moholdt et al., 2024).

It is therefore reasonable for practitioners to (i) document menstrual and menopausal status, (ii) monitor adaptation rather than assume a fixed timeline, and (iii) individualize when objective data indicate it—but recommending that women categorically curtail high‐intensity central‐adaptation training at 3–6 months would outrun the evidence. Current practice guidance is best framed as “monitor and individualize,” not “prescribe by sex.” Table 2 summarizes how these interpretations apply across populations, graded by the strength of the supporting evidence.

**TABLE 2:** Practice‐relevant interpretation by population, graded by evidence strength.

Population/context	Best‐supported interpretation	Evidence	Key references
Sedentary adults, both sexes	Convective delivery predominates; central‐adaptation stimulus (intervals) appropriate initially	Established	(Mandić et al., 2022, 2023; Rosenblat et al., 2022)
Trained men (20–50 years)	Progressive central remodeling supports sustained cardiac‐focused stimulus	Emerging	(Cotie et al., 2025; Howden et al., 2015)
Trained women (20–50 years)	Possible earlier central plateau; monitor rather than prescribe a fixed transition	Emerging (single small cohort)	(Eriksson et al., 2024; Howden et al., 2015)
Elite athletes (VO_2_max >70)	Screen for EIAH; pulmonary limitation plausible; sex difference unresolved	Established (EIAH)/unresolved (sex)	(Dempsey & Wagner, 1999; Hind et al., 2025)
Postmenopausal women	Adaptation modified by estrogen loss; emphasize monitoring; HIIT efficacious	Emerging	(Debray et al., 2023; Miller et al., 2025; Nio et al., 2020)
Women in clinical rehabilitation	Risk of under‐dosing intensity; optimize prescription	Moderate (RCT)	(Khadanga et al., 2022)

*Note*: Recommendations for low and emerging rows are illustrative and provisional, pending validation; they describe interpretive emphasis, not validated protocols.

Abbreviations: EIAH, exercise‐induced arterial hypoxemia; HIIT, high‐intensity interval training; RCT, randomized controlled trial; VO_2_max, maximal oxygen uptake; y, years.

## 7 CURRENT EVIDENCE GAPS AND FUTURE RESEARCH NEEDS

### 7.1 Replication of the central‐adaptation timeline

Multi‐protocol, multi‐site cohorts with serial cardiac imaging (target *n* ≥ 50 per sex per age decade) to test whether the ~3‐month female LV plateau—observed in only 5 women in the original trial (Howden et al., 2015)—generalizes; such studies should retain the gold‐standard imaging of the original while achieving the statistical power it lacked.

### 7.2 A direct test of timing‐based prescription

A sex‐stratified randomized controlled trial in which women complete an initial central‐adaptation phase with imaging‐confirmed plateau detection, then randomize to continued high‐intensity versus peripheral‐focused training, comparing VO_2_max change at ~9 months (≥ 80 women).

### 7.3 Sex and EIAH

Prospective cohorts with arterial sampling and balanced sex distribution, controlling for body size and lung volume, to reconcile Harms et al. (1998) with Hind et al. (2025).

### 7.4 Individualized phenotyping of the limiting step

Whether the rate‐limiting step can be reliably identified in an individual—and whether phenotype‐matched training outperforms guideline‐based prescription—remains an open and testable question. Any such framework faces two prerequisites that are not yet met: prospective validation of classification thresholds (current non‐invasive estimates of cardiac output and arteriovenous oxygen difference carry error margins comparable to the decision intervals they would define), and randomized comparison against standard prescription. Until both are satisfied, individualized phenotyping is best regarded as a hypothesis‐generating research program rather than a clinical tool, with biological sex treated as one modifying covariate among many rather than a deterministic selector.

### 7.5 Structural and reporting priorities

Minimum 50% female enrollment with mandatory sex‐stratified, adequately powered analyses (Elliott‐Sale et al., 2021; Hunter et al., 2023); standardized reporting of menstrual, contraceptive, and menopausal status (D'Souza et al., 2023); routine normalization to fat‐free mass (Kontro et al., 2024); and inclusion of understudied groups (adolescents, adults >75 years, the menopause transition, and individuals on gender‐affirming hormone therapy).

## 8 CONCLUSION

The long‐standing question of whether VO_2_max is centrally or peripherally limited is best answered conditionally: the rate‐limiting step moves along the oxygen transport cascade with training status, modality, environment, age, and—plausibly—biological sex. The contextual determinants are well established; the sex‐specific claims are graded. Sex differences in the magnitude of central remodeling are supported by meta‐analysis; the timeline difference rests on a single small controlled cohort (*n* = 12) and awaits replication; peripheral‐dominant adaptation and sex differences in EIAH remain unresolved, with recent direct evidence finding no EIAH difference between sexes. The epidemiological observation that women achieve equivalent survival at lower fitness is robust, but its mechanistic interpretation is not settled. The defensible conclusions are to adopt sex‐stratified VO_2_max reference standards and to individualize training by monitoring adaptation rather than by applying fixed sex‐based timelines. Identifying and targeting an individual's rate‐limiting step is a promising research direction, but one whose thresholds and prescriptive value require prospective and randomized validation before clinical use. The field's most pressing need is therefore not a new conceptual framework but better‐powered, sex‐stratified, and methodologically standardized evidence—and the explicit grading of that evidence, as attempted here, to keep practice aligned with what is actually known.

## AUTHOR CONTRIBUTIONS

A.R. and S.N. conceived the review and performed the literature appraisal, evidence grading, and drafting of the manuscript. E.P. contributed to critical revision and review of the manuscript for important intellectual content. All authors read and approved the final version of the manuscript and agree to be accountable for all aspects of the work.

## FUNDING INFORMATION

No external funding was received for this work.

## CONFLICT OF INTEREST STATEMENT

The authors declare no conflicts of interest.

## ETHICS STATEMENT

Not applicable. This narrative review synthesizes previously published literature and did not involve new studies of human participants or animals; ethical approval and informed consent were therefore not required.

## Data Availability

Not applicable. This narrative review synthesizes previously published data, with all sources cited in the references.
